# Proof-of-concept prototype development of the self-propelled capsule system for pipeline inspection

**DOI:** 10.1007/s11012-017-0801-3

**Published:** 2017-11-22

**Authors:** Yao Yan, Yang Liu, Joseph Páez Chávez, Florent Zonta, Azat Yusupov

**Affiliations:** 1 School of Aeronautics and Astronautics, University of Electronic Science and Technology of China, Chengdu, 611731 China; 2 College of Engineering Mathematics and Physical Sciences, University of Exeter, Rennes Drive, Exeter, EX4 4RN UK; 3 Center for Applied Dynamical Systems and Computational Methods (CADSCOM), Faculty of Natural Sciences and Mathematics, Escuela Superior Politécnica del Litoral, P.O. Box 09-01-5863, Guayaquil, Ecuador; 4 Center for Dynamics, Department of Mathematics, TU Dresden, 01062 Dresden, Germany; 5 Electrical Engineering, Blaise Pascal University, Clermont-Ferrand, France; 6 School of Engineering, Robert Gordon University, Garthdee Road, Aberdeen, AB10 7GJ UK

**Keywords:** Vibro-impact, Capsule system, Non-smooth dynamical system, Self-propulsion, Pipeline inspection

## Abstract

This paper studies the prototype development for the self-propelled capsule system which is driven by autogenous vibrations and impacts under external resistance forces. This project aims for proof-of-concept of its locomotion in pipeline environment in order to mitigate the technical complexities and difficulties brought by current pressure-driven pipeline inspection technologies. Non-smooth multibody dynamics is applied to describe the motion of the capsule system, and two non-smooth nonlinearities, friction and impact, are considered in modelling. The prototype of the self-propelled capsule system driven by a push-type solenoid with a periodically excited rod has been designed to verify the modelling approach. The prototype contains a microcontroller, a power supply, and a wireless control module, which has been tested in a clear uPVC pipe via remote control. Various control parameters, e.g. impact stiffness, frequency and amplitude of excitation, are studied experimentally, and finally, the fastest progression of the system is obtained.

## Introduction

The development of in-service pipe maintenance for both piggable and unpiggable pipelines is an ongoing challenge in the oil and gas industry [1–3]. Pipeline inspection gauge (known as PIG) is used for various maintenance operations, e.g. cleaning and inspecting pipelines for preventing leaks, physical separation between different fluids flowing through pipeline, or capturing and recording geometric information relating to pipelines. In general, PIG is driven by product flow, and any cause of reduction of the flow, e.g. excessive corrosion or debris within aged pipe, may cause pigging operation failure. A promising solution to overcome this issue is to employ a self-propelled PIG having forward and backward motion control, and with such a rectilinear motion, long-term, remote-controlled inspection becomes feasible. The principle of this solution pioneered by Chernous’ko [4] is that the forward and backward progression of a system in the presence of dry friction can be obtained using a periodically driven inner mass interacting with the main body of the system. The merit of such design is that the system can be encapsuled [5] and self-propelled without the help of additional driving mechanisms (e.g. legs and wheels [6]). This paper will prove this idea by developing a prototype aiming for pipeline inspection. Thus, the main contributions of this paper are to verify the mathematical model of the capsule system studied by Liu et al. [7], demonstrate its rectilinear motion within a dry pipeline experimentally, implement remote control of the prototype, and improve the average speed of the capsule under various control parameters, including impact stiffness, the frequency and amplitude of excitation.

Chernous’ko’s original idea has been followed and extended by many researchers. For example, vibration-induced motion has been discussed in several classical studies by Blekhman [8, 9]. A three-module vibration-driven robot moving on a surface with non-symmetry Coulomb friction was analysed by Fang and Xu [10], who adopted the average method to investigate and optimize the average steady-state velocity of the system. By using elliptical gears, pairs of counterrotating masses and a special design of the stern, Muscia [11] successively drove a hull to move forward without using propellers. Bolotnik et al. [12] proposed a two-body limbless locomotor via linking up two masses using an actuated prismatic joint. From both theoretical and experimental analyses, they have demonstrated that the system can move forward providing that the two bodies of the system have different masses, and the time period for increasing and decreasing the distance between the two bodies are distinct. A box-like robot, which were subjected to the Coulomb dry friction and nonholonomic constrains, containing two inner masses moving orthogonally, was designed by Zhan *et al*. [13]. The robot was implemented two-dimensional motions on a plane, such as translation and rotation without sideslips. Considering all these examples, it can be found that an effective method for implementing rectilinear motion is to introduce asymmetry in system. The systems studied by Fang and Xu [10] and Zhan et al. [13] contain asymmetry in their frictional resistances, and the ship stern [11] and the limbless locomotor [12] introduce their asymmetries through mass distribution.

This paper studies the vibro-impact capsule system which has asymmetry by introducing one-sided internal impacts, i.e. the internal oscillating mass intermittently contacts a displacement constraint leading to bidirectional motion of the entire system. Since this method is effective and practically viable, it has been adopted by many researchers. For example, Nagy et al. [14] designed a microrobot which consists of a main body experiencing the external friction from the supporting surface and the impacts by a hammer driven by wireless oscillating magnetic fields. By modulating the frequency of oscillation, the robot can move forward or backward. This design was then modified by Tung et al. [15] for transporting micropolystyrene and microglass beads in both dry and wet environments. Thereafter, an alternative design, the vibro-impact capsule system, was proposed by Liu et al. [7], which installs a vibro-impact oscillator [16] in the capsule and uses the non-smooth nature for progression. Control parameters, such as mass ratio, frictional forces, and frequency and amplitude of excitation, were fully studied for improving its forward and backward motion [17]. A preliminary experimental study has been carried out by Liu et al. [18], and the comparison between numerical and experimental results showed a good agreement. More recently, a position feedback controller was employed for the capsule system to give rise to a dynamical scenario with two coexisting solutions corresponding to forward and backward progression [19]. Thus, direction of progression of the capsule can be controlled by suitably perturbing its initial conditions, without altering control parameters. In this paper, the prototype of the vibro-impact capsule system was developed with all modules encapsuled in a single unit, including a linear actuator, a control board, an energy source, and a wireless communication module.

The contribution of this paper is to develop the proof-of-concept prototype of the self-propelled capsule system, aiming to demonstrate the numerical studies conducted by Liu et al. [7] in a realistic environment. Unlike the wheeled and legged robots, e.g. [20, 21], the proposed capsule system can be encapsuled without any external driving mechanism, which is more suitable to be used in complex environment. Although the prototype has not been tested in field, the results presented here preliminarily demonstrate the concept. The rest of this paper is organized as follows. Section 2 introduces the mathematical model of the capsule system, and numerical results are given to study the non-smooth nature of the system. In Sect. 3, both mechanical and electrical designs of the prototype are described in detail followed by parameter identification and experimental investigation in Sect. 4. Finally, in Sect. 5, some conclusions are drawn.

## Mathematical modelling

### Equations of motion

**Fig. 1:**
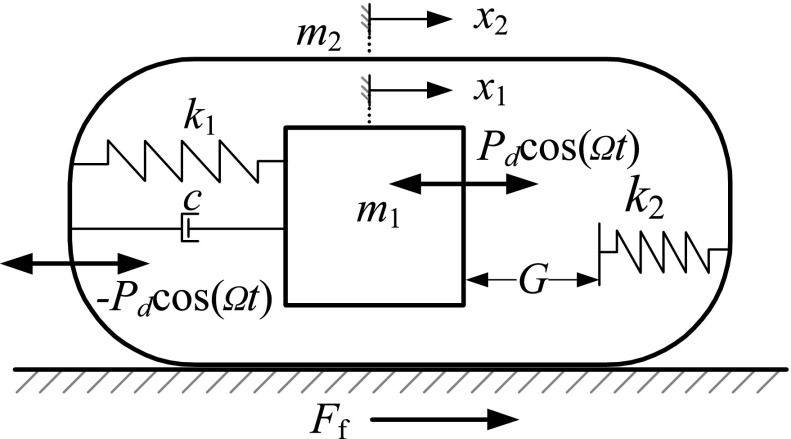
Physical model of the self-propelled capsule

The vibro-impact capsule model considered in this work can be seen in Fig. 1, which shows the mechanical setup with two degrees of freedom. The system consists of a movable rod $m_{1}$ attached to a main body $m_{2}$ via a spring of stiffness $k_{1}$ and a damper with coefficient *c*. The movable rod is excited periodically via a push-type solenoid driven by a sinusoidal signal, with amplitude $P_{d}$ and angular frequency $\Omega$. According to Newton’s third law, there is a counter force equally in magnitude and oppositely in direction acting on the capsule. When the amplitude of oscillation of the rod is sufficiently large, impacts between the rod and a weightless plate take place, where the plate is assumed to be connected to the main body via a linear spring with stiffness $k_{2}$. In addition, the model considers a Coulomb resistance force $F_{f}=-{\text {sign}}(\dot{x}_2)P_f$ from environment. Here, $P_f=\mu (m_1+m_2)g$, where $\mu$ is the coefficient of friction and *g* stands for the acceleration due to gravity. As this paper only experimentally validates the mathematical model using a dry pipe, Coulomb friction is appropriate to reflect the contact between the capsule and the internal wall of the pipe. However, in fluid environment, the condition becomes more complicated, and it is difficult to model the fluid resistance on capsule comprehensively. Preferable approach is to use the Computational Fluid Dynamics (CFD) analysis with consideration of fluid lift and drag coefficients. A detailed CFD analysis of the capsule system can be found in [17].

In the present work, we will denote by $z:=(x_{1},x_{2},y_{1},y_{2})^T\in {{\mathrm{\mathbbm {R}}}}^4$ and $\alpha :=(\Omega ,P_{d},c,k_{1},k_{2},m_{1},m_{2},\mu ,$ $G)\in \left( {{\mathrm{\mathbbm {R}}}}^+\right) ^9$ the state variables and parameters of the system, respectively, where ${{\mathrm{\mathbbm {R}}}}^+$ stands for the set of positive numbers. According to the mechanical setup described above, the system can operate under two modes, *Contact* and *No contact*, depending on whether the rod is in contact or not with the secondary spring $k_{2}$. The equations describing the motion of the system can be written in compact form as follows (cf. [19]):

*No contact* ($$x_{1}-x_{2}<G$$):

1 $$\begin{aligned} \dot{z}={\left\{ \begin{array}{ll} f_{\text {NC-S}}(t,z,\alpha ), {}\,\,\, \dot{x}_{2}=0 \text{ and } |k_{1}(x_{1}-x_{2})+c(\dot{x}_{1}-\dot{x}_{2}) -P_{d}\cos \left( \Omega t\right) |\le P_f,\\ f_{\text {NC-FD}}(t,z,\alpha ), {}\,\,\, \dot{x}_{2}>0 \text{ or } \left( \dot{x}_{2}=0 \text{ and } k_{1}(x_{1}-x_{2})+c(\dot{x}_{1}-\dot{x}_{2})-P_{d}\cos \left( \Omega t\right) >P_f\right) ,\\ f_{\text {NC-BD}}(t,z,\alpha ), {}\,\,\, \dot{x}_{2}<0 \text{ or } \left( \dot{x}_{2}=0 \text{ and } k_{1}(x_{1}-x_{2})+c(\dot{x}_{1}-\dot{x}_{2})-P_{d}\cos \left( \Omega t\right) <-P_f\right) . \end{array}\right. } \end{aligned}$$

*Contact* ($$x_{1}-x_{2}\ge G$$):

2 $$\begin{aligned} \dot{z}={\left\{ \begin{array}{ll} f_{\text {C-S}}(t,z,\alpha ), &{}\quad \dot{x}_{2}=0 \text{ and } |{k_{1}(x_{1}-x_{2})+c(\dot{x}_{1}-\dot{x}_{2})+ k_{2}(x_{1}-x_{2}-G)-P_{d}\cos \left( \Omega t\right) }|\le P_f,\\ f_{\text {C-FD}}(t,z,\alpha ), &{}\,\,\, \dot{x}_{2}>0 \text{ or } \left( \dot{x}_{2}=0 \text{ and } k_{1}(x_{1}-x_{2})+c(\dot{x}_{1}-\dot{x}_{2})+ k_{2}(x_{1}-x_{2}-G)\right. \\ &{}\left. -P_{d}\cos \left( \Omega t\right) \right) >P_f,\\ f_{\text {C-BD}}(t,z,\alpha ), &{}\,\,\, \dot{x}_{2}<0 \text{ or } \left( \dot{x}_{2}=0 \text{ and } k_{1}(x_{1}-x_{2})+c(\dot{x}_{1}-\dot{x}_{2})+ k_{2}(x_{1}-x_{2}-G)\right. \\ &{}\left. -P_{d}\cos \left( \Omega t\right) <-P_f\right) , \end{array}\right. } \end{aligned}$$

where $G>0$ stands for the gap between the mass and the secondary spring in the equilibrium position, see Fig. 1. As can be seen from the equations above, both the *Contact* and *No contact* regimes contain three submodes. The first one corresponds to the case when the capsule is not moving, while the second and third submodes occur when the capsule moves forward and backward, respectively. In this framework, the corresponding vector fields are defined as follows (cf. [19]):

Capsule not moving ($\dot{x}_{2}=0$):

$$\begin{aligned} f_{\text {NC-S}}(t,z,\alpha )&:=\left( \begin{array}{c} y_{1}\\ 0\\ \frac{1}{m_{1}}\left( P_{d}\cos \left( \Omega t\right) +k_{1}(x_{2}-x_{1})+c(y_{2}-y_{1})\right) \\ 0 \end{array}\right) ,\\ f_{\text {C-S}}(t,z,\alpha )&:=\left( \begin{array}{c} y_{1}\\ 0\\ \frac{1}{m_{1}}\left( P_{d}\cos \left( \Omega t\right) +k_{1}(x_{2}-x_{1})+c(y_{2}-y_{1})-k_{2}(x_{1}-x_{2}-G)\right) \\ 0 \end{array}\right) . \end{aligned}$$

Capsule moving forward ($\dot{x}_{2}>0$):

$$\begin{aligned} f_{\text {NC-FD}}(t,z,\alpha )&:=\left( \begin{array}{c} y_{1}\\ y_{2}\\ \frac{1}{m_{1}}\left( P_{d}\cos \left( \Omega t\right) +k_{1}(x_{2}-x_{1})+c(y_{2}-y_{1})\right) \\ \frac{1}{m_{2}}\left( -P_{d}\cos \left( \Omega t\right) -k_{1}(x_{2}-x_{1})-c(y_{2}-y_{1})-P_f\right) \end{array}\right) ,\\ f_{\text {C-FD}}(t,z,\alpha )&:=\left( \begin{array}{c} y_{1}\\ y_{2}\\ \frac{1}{m_{1}}\left( P_{d}\cos \left( \Omega t\right) +k_{1}(x_{2}-x_{1})+c(y_{2}-y_{1})-k_{2}(x_{1}-x_{2}-G)\right) \\ \frac{1}{m_{2}}\left( k_{2}(x_{1}-x_{2}-G)-P_{d}\cos \left( \Omega t\right) -k_{1}(x_{2}-x_{1})-c(y_{2}-y_{1})-P_f\right) \end{array}\right) . \end{aligned}$$

Capsule moving backward ($\dot{x}_{2}<0$):

$$\begin{aligned} f_{\text {NC-BD}}(t,z,\alpha )&:=\left( \begin{array}{c} y_{1}\\ y_{2}\\ \frac{1}{m_{1}}\left( P_{d}\cos \left( \Omega t\right) +k_{1}(x_{2}-x_{1})+c(y_{2}-y_{1})\right) \\ \frac{1}{m_{2}}\left( P_f-P_{d}\cos \left( \Omega t\right) -k_{1}(x_{2}-x_{1})-c(y_{2}-y_{1})\right) \end{array}\right) ,\\ f_{\text {C-BD}}(t,z,\alpha )&:=\left( \begin{array}{c} y_{1}\\ y_{2}\\ \frac{1}{m_{1}}\left( P_{d}\cos \left( \Omega t\right) +k_{1}(x_{2}-x_{1})+c(y_{2}-y_{1})-k_{2}(x_{1}-x_{2}-G)\right) \\ \frac{1}{m_{2}}\left( k_{2}(x_{1}-x_{2}-G)-P_{d}\cos \left( \Omega t\right) -k_{1}(x_{2}-x_{1})-c(y_{2}-y_{1})+P_f\right) \end{array}\right) . \end{aligned}$$

To conclude this section, let us point out that the expression $F_{f}=-{\text {sign}}(\dot{x}_2)P_f$ used to model Coulomb friction is only valid for $\dot{x}_{2}\ne 0$. For $\dot{x}_{2}=0$, the capsule is in stationary position, and hence the friction adjusts itself to enforce the equilibrium with the remaining forces acting on the capsule, according to Newton’s third law. This is considered in the vector fields $f_{\text {NC-S}}$ and $f_{\text {C-S}}$ (see above), where the second and fourth components are zero, reflecting the fact that both velocity and acceleration of the capsule are zero during the stationary regime.

## Preliminary numerical study of the capsule response

In order to study the behaviour of the self-propelled capsule system, we will employ two different types of numerical technique, namely, direct numerical integration and path-following methods (via the continuation platform COCO [22]). As was explained in the preceding section, system (1)–(2) belongs to the class of piecewise-smooth dynamical systems, which are characterized by periods of smooth evolution interrupted by instantaneous events. Typically, in these systems the state space is divided into disjoint subregions, in such a way that the system dynamics in each region is described by a smooth vector field. Therefore, special care must be taken in order to get reliable numerical approximations of the behavior of such systems in an efficient way. In our investigation, the numerical simulations will be obtained via direct numerical integration of one of the possible smooth vector fields (as specified in the previous section), until the computed solution approaches the boundary of the corresponding region (for example, the impact boundary $x_{1}-x_{2}=G$). The boundary point is accurately detected and then the integrated vector field is switched according to the governing laws of the system (1)–(2). In the present work, this will be implemented by means of the standard MATLAB ODE solvers together with their built-in event location routines [23, 24], as suggested in [25]. Finally, all numerical computations are carried out with the default error tolerances provided by the MATLAB ODE solvers and the the continuation package COCO.

**Fig. 2:**
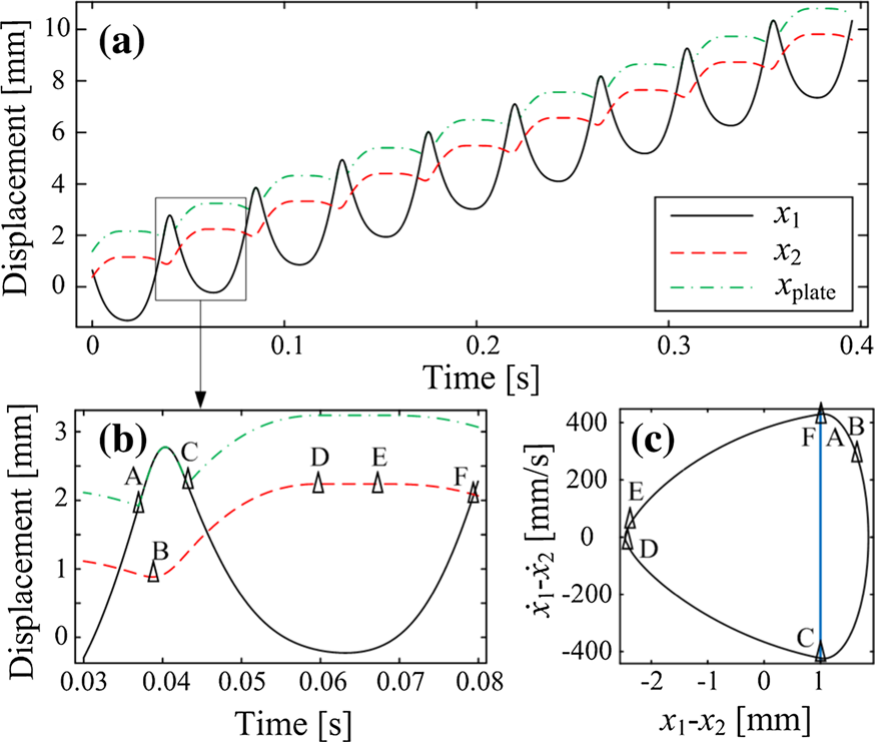
**a** Forward progression of the capsule obtained for a period-one motion of the capsule system with one impact per period of excitation. The displacements of the inner mass, the capsule, and the weightless plate are plotted in black, red, and green, respectively. **b** Blow-up of one period of motion and **c** its corresponding phase plot. The location of the impact boundary is shown by the vertical blue line. (Color figure online)

According to the mechanical configuration described in the previous section, the vibro-impact capsule system can move in two directions: forward and backward. The direction of motion of the capsule is determined by its structural parameters, e.g. mass ratio $m_1/m_2$, stiffness ratio $k_1/k_2$, as well as the external sinusoidal excitation, controlled by the parameters $\Omega$ (frequency) and $P_d$ (amplitude). Figure 2 presents a period-one response of the capsule with forward progression, computed for the parameter values $m_1=0.1$ kg, $m_2=0.4$ kg, $k_1=1.5\times 10^{3}$ N/m, $k_2=2.2\times 10^{4}$ N/m, $c=0.01$ Ns/m, $P_d=3$ N, $\omega =140$ rad/s, $G=1$ mm and $\mu =0.3$.

As can be seen from Fig. 2, the depicted period-one response operates under the regimes *Contact* ($x_{1}-x_{2}\ge G$) and *No contact* ($x_{1}-x_{2}<G$) introduced previously. The behavior of the position of the capsule with respect to the time is shown in panel (b), for exactly one period of external excitation, while a phase plot of the periodic solution is presented in panel (c). In these diagrams, some special points are marked for a better understanding of the system response. The point labeled A in Fig. 2b, c marks the transition from *No contact* to *Contact*, where an impact with the secondary spring $k_{2}$ (plate) occurs, while the capsule is moving backward. At point B, shortly after the impact, the capsule changes direction, and the device moves forward thereafter. The mass $m_{1}$ separates from the plate at point C, after which the system operates under the *No contact* regime. During this mode the velocity of the capsule decreases, until it attains a stationary position at D. The capsule remains so until the point E is reached, where the force acting on the capsule is large enough to overcome the dry friction threshold, and the capsule starts moving in the backward direction. This motion continues until the point F is reached, where the cycle described above repeats itself.

**Fig. 3:**
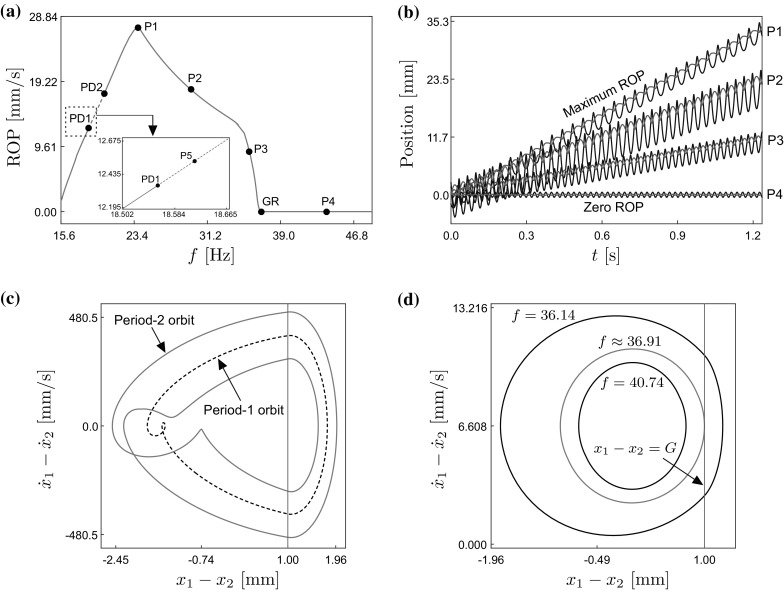
**a** Numerical continuation of the periodic orbit shown in Fig. 2c with respect to the frequency of excitation $f=\frac{1}{2\pi }\Omega$, computed for the parameter values $m_1=0.1$ kg, $m_2=0.4$ kg, $k_1=1.5\times 10^{3}$ N/m, $k_2=2.2\times 10^{4}$ N/m, $c=0.01$ Ns/m, $P_d=3$ N, $G=1$ mm and $\mu =0.3$. The points GR and PD*i* represent grazing and period-doubling bifurcations of limit cycles, respectively, while the labels P*i* denote test points along the bifurcation diagram. The inner plot shows a blow-up of the bifurcation diagram around the period-doubling bifurcation PD1. **b** Time histories of the position of the mass $m_{1}$ ($x_{1}$, black) and the capsule ($x_{2}$, red), computed at the test points P*i* shown on panel **a**. **c** Phase plot of the period-two (blue, stable) and period-one (black, unstable) solutions computed at the test point P5. **d** Sequence of periodic orbits computed near the grazing bifurcation GR ($f\approx 36.91$). The vertical line shown in panels **c** and **d** stands for the discontinuity boundary $x_{1}-x_{2}=G$, which defines the transition between *Contact* and *No contact* modes. (Color figure online)

Next, we will carry out a preliminary numerical study of the capsule system via path-following techniques for non-smooth dynamical systems. Specifically, we will apply the continuation platform COCO [22] to carry out the numerical continuation of the period-one response shown in Fig. 2c with respect to the frequency of excitation $f=\frac{1}{2\pi }\Omega$. For this purpose, it is convenient to introduce the following coordinate transformation (cf. [26])

3 $$\begin{aligned} \left\{ \begin{aligned} w_{1}&=x_{1},\\ w_{2}&=x_{1}-x_{2},\\ v_{1}&=y_{1},\\ v_{2}&=y_{1}-y_{2}. \end{aligned}\right. \end{aligned}$$

This is a linear variable change that allows decoupling the periodic component of the capsule response from the drift, in such a way that continuation methods can be applied to study the oscillatory behavior of the system separately. In (3), the new variables $w_{2}$ and $v_{2}$ represent the position and velocity of the internal mass $m_{1}$ with respect to the position and velocity of the capsule, respectively.

After applying the coordinate transformation (3), the numerical continuation of the periodic orbit shown in Fig. 2c can be carried out via the continuation platform COCO. The result can be seen in Fig. 3. Panel (a) presents the resulting bifurcation diagram obtained via COCO, showing the behavior of the rate of progression (ROP) with respect to the driving frequency *f* of the sinusoidal excitation. The analysis reveals critical frequency values for which the period-one orbit loses stability. In this diagram, solid and dashed lines are used so as to distinguish stable and unstable period-one solutions, respectively. As can be seen in panel (a), for low frequency values there is a branch of stable period-one orbits for which the ROP increases with the driving frequency. This branch is interrupted at the point labeled PD1 ($f\approx 18.5570$ Hz), where the periodic solution undergoes a supercritical period-doubling bifurcation. After this point, the period-one solution becomes unstable and a family of stable period-two solutions is born. These solutions can be seen in Fig. 3c, computed a the test point $f=18.62$ Hz (labeled P5). If the frequency is further increased, another supercritical period-doubling bifurcation is found for $f\approx 20.2254$ Hz (PD2), where the period-one solution regains stability and the family of period-two orbits disappears.

After the bifurcation PD2, the ROP continues increasing with the frequency, until the critical parameter value $f\approx 23.7924$ Hz (point P1) is encountered, where the ROP attains a maximum. Figure 3b presents the system response computed at P1, as well as various additional test points $f=29.43$ Hz (P2), $f=35.67$ Hz (P3) and $f=43.86$ Hz (P4). As can be seen in panel (b), the ROP varies from zero (at P4) to approximately 27.18 mm/s (at P1), where the ROP achieves a maximum. If the frequency is increased beyond the maximum P1, the ROP presents a decreasing behavior, and becomes zero at the critical point GR ($f\approx 36.9105$ Hz), where the period-one orbit undergoes a grazing bifurcation. Figure 3d presents the periodic response of the system before, at and after the grazing bifurcation. Further increments of the driving frequency produces no additional relevant dynamical phenomena, while the ROP remains zero.

This preliminary numerical study reveals some of the dynamical features of the considered capsule system, which provide valuable information for the design of the prototype and the experimental tests. The subsequent sections of this work will be dedicated to the specific development and construction of the capsule system, as well as the detailed experimental studies based on this model.

## Prototype development

### Capsule design

**Fig. 4:**
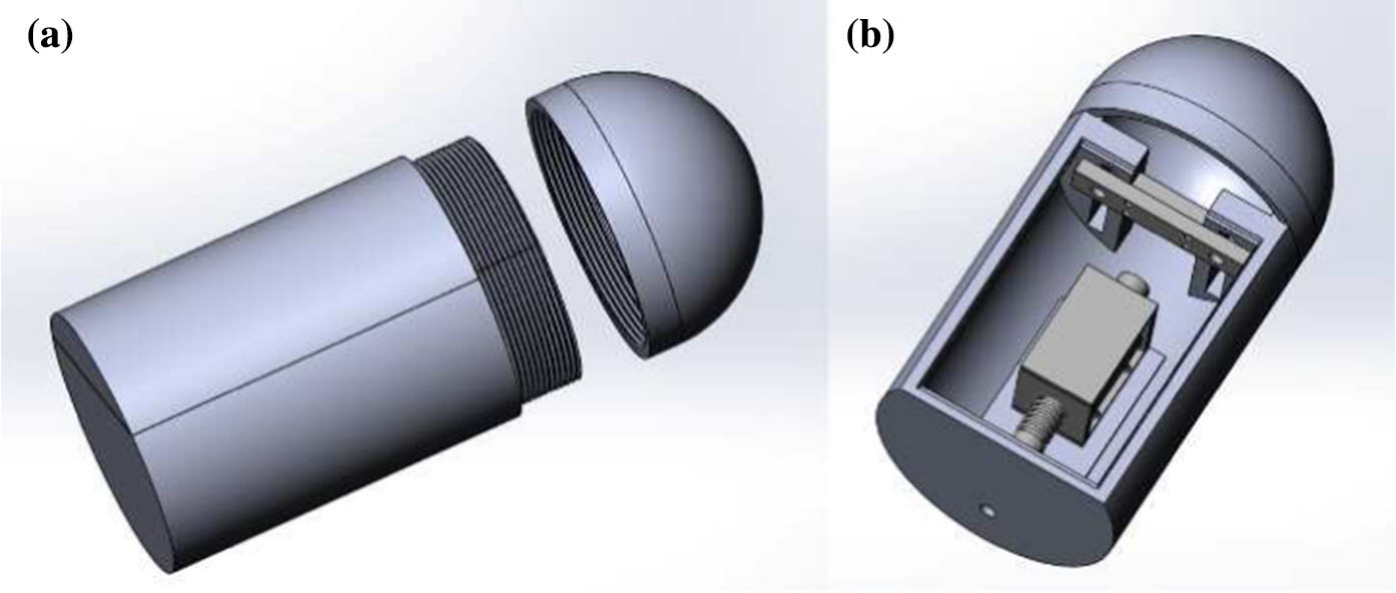
Prototype design of the vibro-impact capsule system [28]

**Fig. 5:**
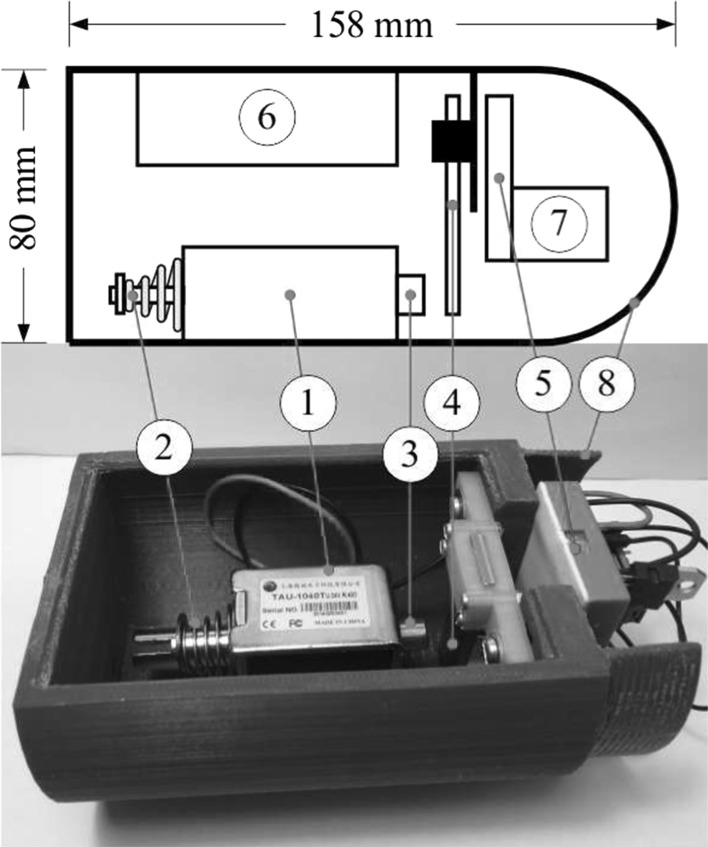
Schematics and photograph of the capsule prototype. The main components are marked as follows: (1) push-pull solenoid, (2) primary spring, (3) rod, (4) steel plate, (5) microcontroller and transistor, (6) power supply for the solenoid, (7) power supply for the controller, and (8) capsule shell

Based on the mathematical model studied above, the prototype of the capsule system was designed using SolidWorks as shown in Fig. 4, and manufactured via 3D printing using plastics. The schematics and the photograph of the prototype are presented in Fig. 5, which the main components of the prototype are encapsuled within a purple shell with a length of 158 mm and a diameter of 80 mm. A 10 mm-throw push-type solenoid with a periodically excited rod is mounted on the capsule. The motion of the rod is restricted by a helical spring (marked by $\textcircled {2}$) connected with the solenoid and a clamped steel plate (shown by $\textcircled {4}$). Here, the helical spring and the steel plate correspond to the primary spring $k_1$ and the secondary spring $k_2$ as shown in Fig. 1, respectively. With the consideration of the elastic modulus, the geometry, and the boundary condition of the plate, the stiffness of the secondary spring was approximated by using [27]

4 $$\begin{aligned} k_2\approx \frac{E d h^3}{4\, l^3}, \end{aligned}$$

where *l* is the length of overhang, *h* is the thickness, *d* is the width, and *E* is the Young’s modulus of the steel plate. By changing the length of the plate *l*, the stiffness of the secondary spring $k_2$ can be varied so that the dynamics of the capsule may be affected leading to different average rates of progression. The detailed studies of capsule dynamics under various control parameters can be found from [7].

### Actuator

The actuation force is generated by a push-type solenoid as shown by $\textcircled {1}$ in Fig. 5, which consists of an electromagnetically inductive coil wound around a movable rod (marked by $\textcircled {3}$) connected with the primary spring (shown by $\textcircled {2}$). When the coil is energized, it generates strong electromagnetic fields to push the rod to move forward. When the power is off, the elastic force provided by the primary spring helps the rod to move back to its original position. The solenoid can be operated within 9-24 V and its coil resistance is 43 $\Omega$. According to the specification of the solenoid, it can exert a force of 10 N over 5 mm stroke when it is powered with 29 V, but a limitation of duty cycle at 50% is required to prevent overheating. In our experiment, the solenoid was supplied with a maximum of 20 V which can generate the maximal force at 5 N over 5 mm stroke. In particular, we applied harmonic excitation to the rod by using digital-to-analog converter (DAC), and the details of this controller will be studied in the next subsection.

### Controller

The original controller of the solenoid has a resolution of 8 bits and runs at 12 MHz, but only has on-off control. In order to generate vibro-impact oscillation on the rod, harmonic control signal is required for the solenoid. Therefore, DAC was used to convert discrete numerical numbers into a continuous tension in voltage. Such a converter is characterized by the quantum governed by $q=\tfrac{V_{pe}}{2^n}$, where $V_{pe}$ is the supply tension and *n* is the resolution. For example, when a 5 V tension is applied on a 8 bits controller, one has the quantum $q=\frac{5}{2^8}=19.5$ mV, which is the smallest increment of the controller.

Using DAC, harmonic signal can be generated by using a sinusoidal wave lookup table, i.e. a period of the signal is divided into several spans, and each span is represented by a specific value. For the other points which are out of the time period, they can be mapped into this period since the signal is periodic. Thus, the controller only needs to execute a small text file, which includes a discrete sinusoidal function in one period. In order to alter the amplitude of the wave, the recorded sinusoid which has an amplitude of 1 needs to multiply a desired value. To set the frequency of the sinusoid, a “delay” function which allows to set the time between the generation of lookup table elements is employed. By applying these modifications, the frequency and amplitude of the sinusoidal wave become adjustable.

**Fig. 6:**
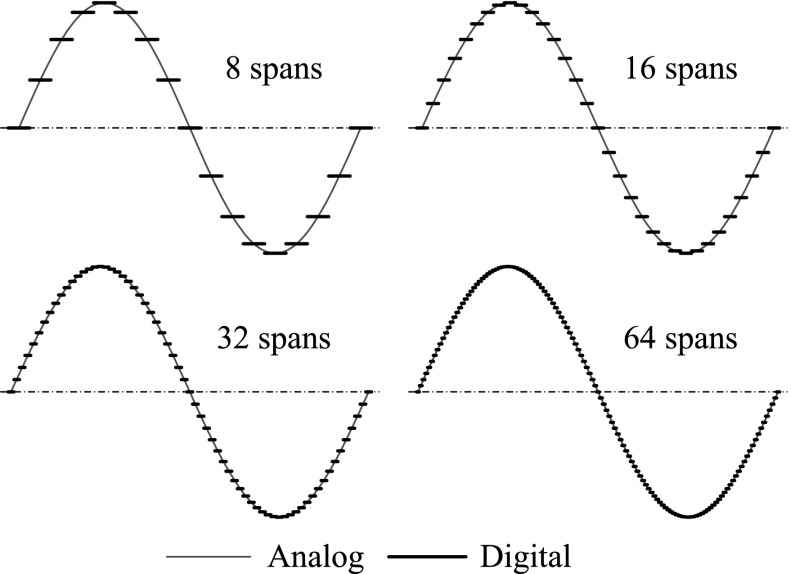
Analog sinusoidal waves (red lines) and their corresponding digital signals (black lines) with 8, 16, 32 and 64 time spans. (Color figure online)

The analog sinusoidal waves and their corresponding digital signals with different time spans are illustrated in Fig. 6. As can be seen from the figure, in order to have a good approximation of the analog sinusoidal wave, the lookup table with more time spans is preferred. For example, the digital with 64 spans in one period has a much better performance than the one with 8 spans. However, the number of spans cannot be increased infinitely due to the requirement of memory space, so the lookup table with less spans requires less memory space and is more flexible for altering the frequency of excitation. The time period of a sinusoid wave is determined by the number of spans and the duration of each span, $T=N \Delta t$, where *T* is the period time, *N* the number of spans and $\Delta t$ is the duration of each span. The duration $\Delta t$ depends on the “delay” function $t_{d}$ and the inherent delay $t_{i}$ introduced by calculation, searching the lookup table, and communication between the controller and the DAC. Therefore, even $t_{d}$ is set to 0, the minimum period time is $T_{min}=N_{ti}$, and the maximum frequency is $f_{max}=\frac{1}{N_{ti}}$. For example, when $t_{d}=5$ ms, the range of the signal frequency was tested and presented in Table 1. From the table, it can be seen that, with the increase of the number of spans, the available frequency range is reduced. In addition, based on our tests, we found that the solenoid stops working when the excitation frequency is above 32 Hz. Finally, considering the smoothness and the frequency of the sinusoidal signal, we chose 64 time spans for our experiments.

**Table 1:** Frequency range in terms of the number of time spans

Number of spans	256	128	64
Minimum frequency (Hz)	< 1	1.4	2.8
Maximum frequency (Hz)	10.2	17.14	34.0

**Fig. 7:**
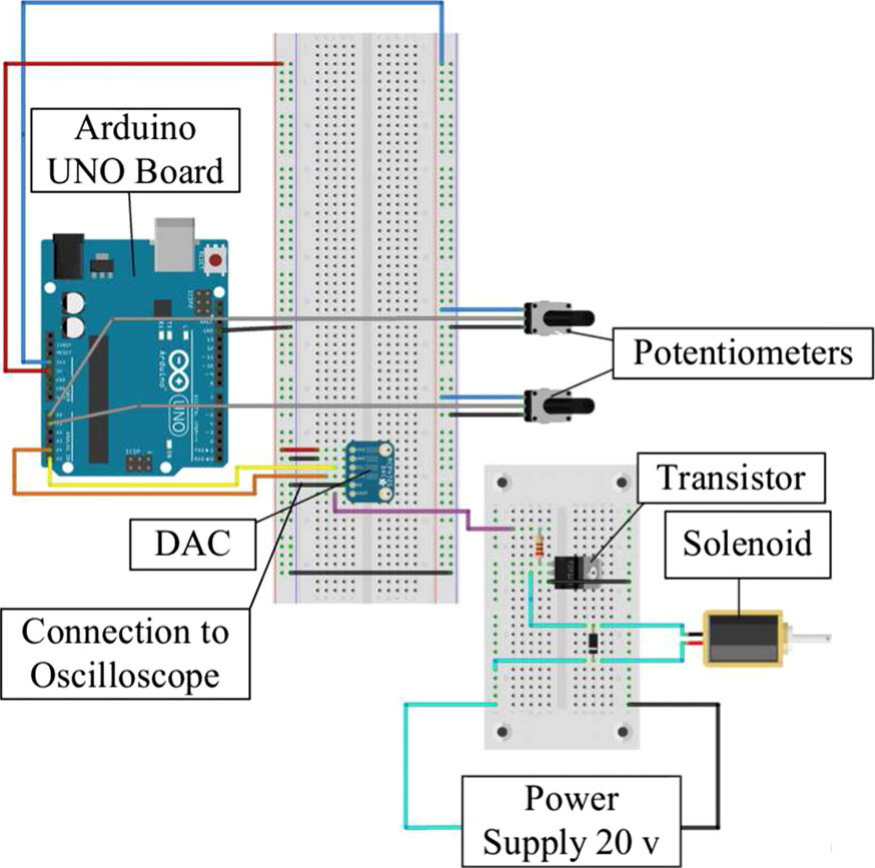
Electric circuit of the controller consisting of two potentiometers, an Arduino UNO board, a DAC, and a transistor

The electric circuit of the controller which was built based on Arduino is presented in Fig. 7. The control parameters, i.e. the amplitude, the frequency and the offset of the sinusoidal signal, are set by potentiometers. The solenoid has a voltage threshold (i.e. the offset) for actuation which means that sufficient current has to pass through the coil in order to oscillate the rod. This was done by connecting a potentiometer to the transistor which can add offset tension to the sinusoidal signal. The control parameters were received by the Arduino UNO board, and transformed into integer values for the sinusoidal signal. Then, this digital signal was converted into analog by the DAC, and thereafter, the analog signal was sent to the transistor for driving the solenoid. The power supply for the Arduino board is 3 V which is below the requirement of the solenoid (i.e. 9–24 V), so an extra power supply consisting of a Darlington transistor and two series connected 9 V batteries was provided for the solenoid.

### Wireless module

Remote control of the capsule system was developed using the radio frequency (RF) wave. Two transceiver RF modules were used to implement the communication between the transmitter and the receiver. The RF transceiver nRF24L01 which consists of an oscillator and an integrated antenna was used, and this device works in serial peripheral interface for communication running at 2.4 GHz with a low power consumption. The nRF24L01 can transmit and receive data in a range of 100 m in an open area using two types of communication protocols, ShockBurst and Enhanced ShockBurst, and 6 data pipes. In our design, the wireless module of the capsule system uses the ShockBurst protocol and pipe 0, running at the frequency of 2 GHz and the data transmitting rate of 250 kbps, with the address of the receiver setting at 0xFFF.

**Fig. 8:**
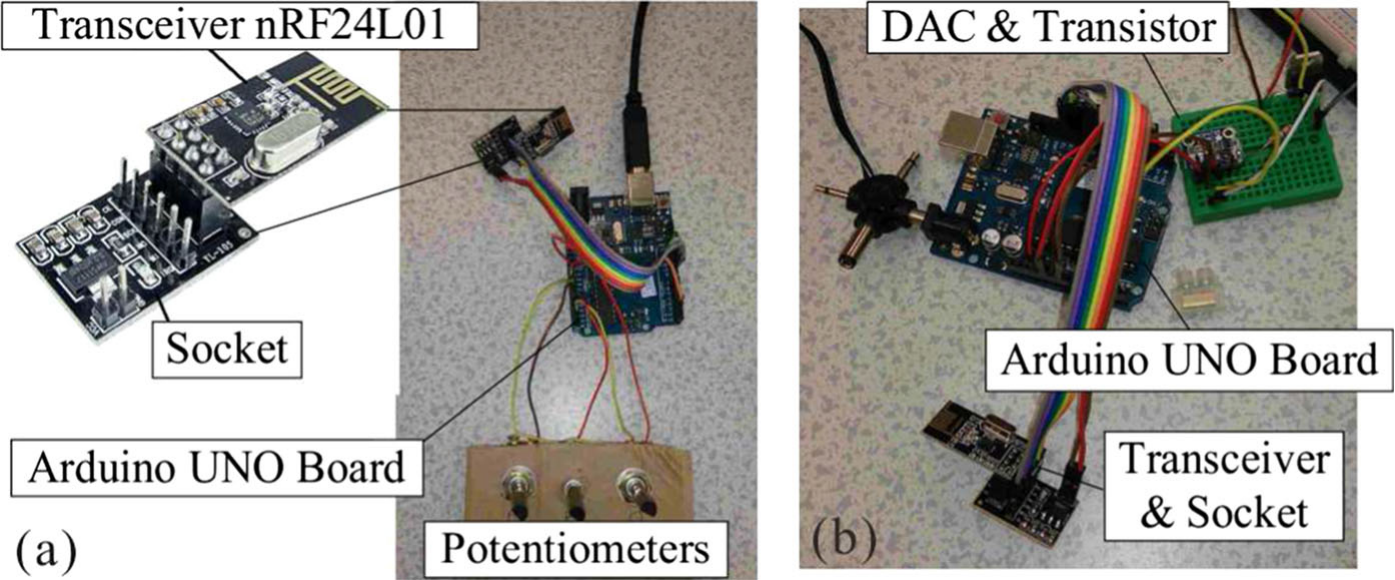
**a** Remote control board, where potentiometers are installed to set the amplitude, the frequency, and the offset of the sinusoidal signal. **b** Command receiver board connected with the capsule system which receives the control parameters sent by the remote control board, and then transforms these parameters into the desired sinusoidal signal for driving the solenoid

Two modules for wireless communication, the remote control board and the command receiver board, are shown in Fig. 8. The command receiver board was connected to the capsule to receive data transmitted from the remote control board. As can be seen from Fig. 8a, the remote control board has three potentiometers for setting the amplitude, the frequency and the offset of the sinusoidal signal, and these data are transferred to the RF transceiver via the Arduino UNO board and the socket. The transceiver transmits these parameter values to another transceiver on the command receiver board presented in Fig. 8b via the RF wave per 100 ms. Once receiving these parameter values, the receiver board can generate analog sinusoidal signal from the DAC and the transistor.

## Experiment and results

### Test bed

**Fig. 9:**
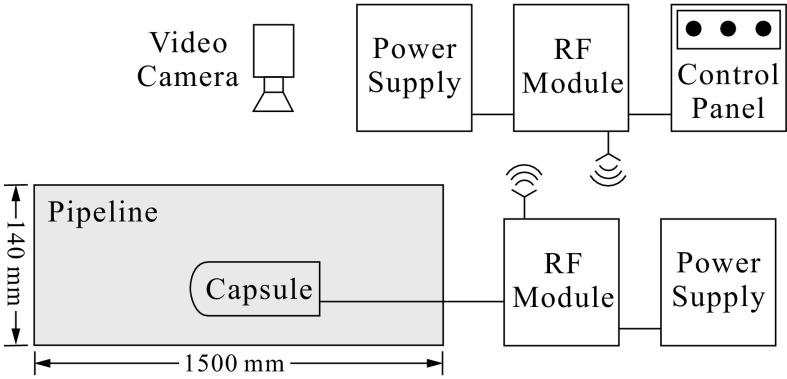
Schematics of the experimental setup

**Fig. 10:**
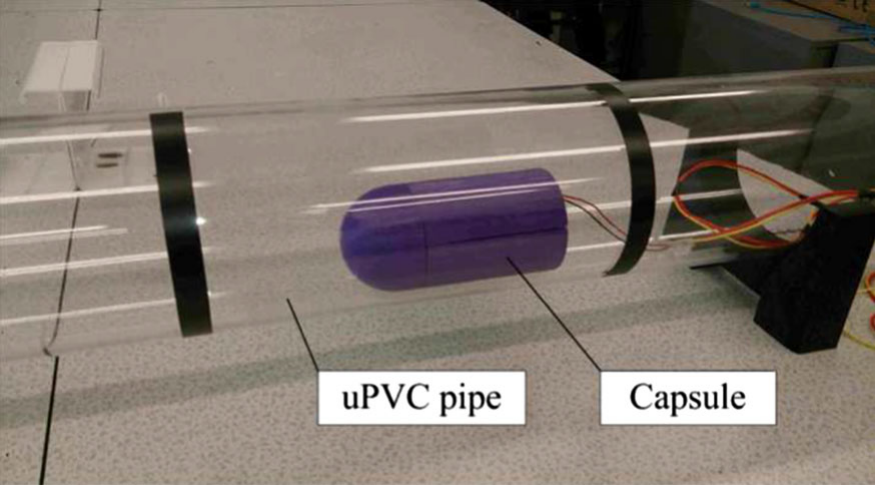
Test bed for the capsule prototype, where the vibro-impact capsule is placed in a transparent uPVC pipe, and the capsule motion is recorded by a video camera at the frame rate of 30 fps

Figure 9 shows the schematics of the experimental rig, where an uPVC clear pipe with a diameter of 140 mm and a length of 1500 mm was used as test bed. The displacement of the capsule was recorded using a video camera with a resolution of 1080 pixels and a frame rate of 30 fps. The photograph of the capsule in the uPVC pipe is present in Fig. 10.

### Parameter identification

The rod of the solenoid which provides the inner mass of the capsule $m_1$ is weighted at 0.03 kg, and the weight of the rest of the system $m_2$ is 0.38 kg. In order to measure the stiffness $k_1$ and the damping coefficient *c* of the primary spring, it was removed from the solenoid and clamped on a test bed with an additional mass attached to its end for free vibration test. Then, the stiffness $k_1$ was determined from the natural frequency of the recorded free vibrations, and the logarithmic decrement method [29] was adopted to determine the damping coefficient *c*. The stiffness of the impact plate $k_2$ was calculated using Eq. 4, and the gap between the rod and the plate was measured once the rod was positioned.

Measurement of the frictional coefficient $\mu$ between the capsule and the pipe was done by both static and dynamic tests. The static test was carried out by placing the capsule and the pipe horizontally, and then lifted one end of the pipe slowly until the capsule started to slide. The inclination angle $\theta$ at this moment was recorded for calculating the static frictional coefficient by using $\mu _{s}=\tan (\theta )$. The dynamic test was carried out by placing both the capsule and the pipe horizontally, and gave the capsule an arbitrary initial velocity $v_0$ by pushing it gently. The dynamic friction coefficient $\mu _{d}$ was then calculated using the energy equivalent equation

5 $$\begin{aligned} \frac{1}{2}(m_1+m_2)v_0^2=\mu _{d}(m_1+m_2)gs, \end{aligned}$$

where $\mu _{d}=\frac{v_0^2}{2gs}$, *s* is the travel distance of the capsule subject to the arbitrary push and the dynamic friction. Both the static and the dynamic tests were run for 5 times, and the average value of these coefficients was adopted for the fictional coefficient in the Coulomb friction model. Detailed descriptions of procedures for both static and dynamic tests can be found from [18].

Finally, the identified physical parameters of the vibro-impact capsule system are given in Table 2, where $P_{v}$ and $P_{o}$ are the amplitude and the offset of the sinusoidal signal in voltage, respectively.

**Table 2:** Identified physical parameters of the prototype

Parameters	Value	Units
$m_1$	0.03	kg
$m_2$	0.38	kg
$k_1$	0.16	kN/m
*c*	0.03	Ns/m
$\mu$	0.5	–
*G*	7	mm
$k_2$	Various	kN/m
$\Omega$	Various	Hz
$P_{v}$	Various	V
$P_{o}$	Various	V

### Numerical and experimental results

This section presents the simulation results obtained by using the mathematical model studied in Sect. 2 and the experimental results carried out by using the experimental test bed. Experiments were run for various control parameters, including the stiffness of the steel plate $k_2$, the frequency, the amplitude, and the offset of the sinusoidal wave, and both forward and backward motions of the prototype were observed.

**Fig. 11:**
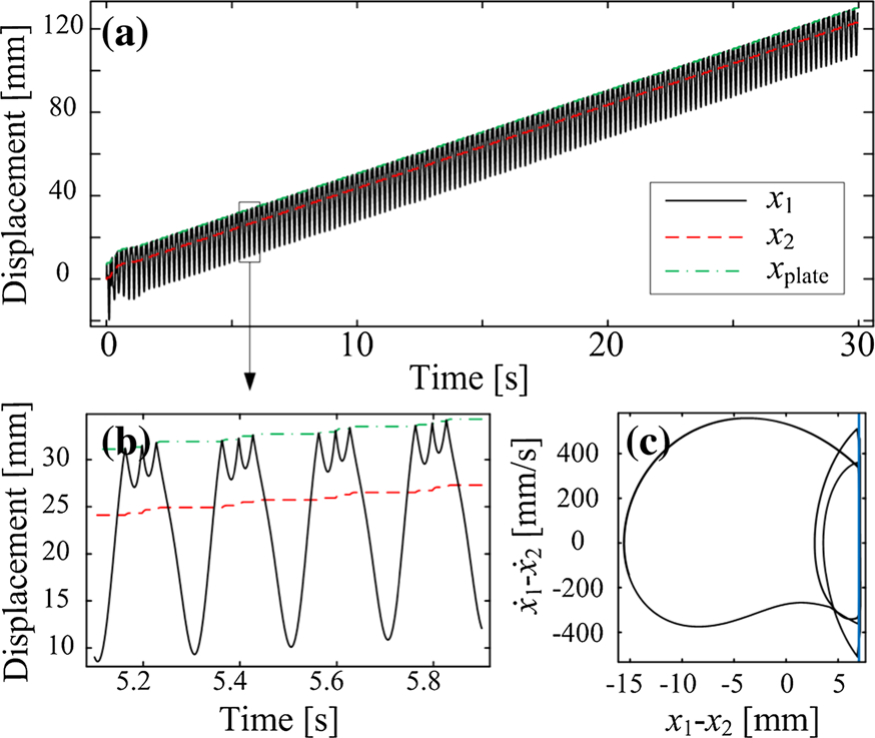
Numerical results: **a** time histories of displacements of the capsule system, **b** zoom up of time histories, and **c** phase trajectory on the phase plane ($x_1$-$x_2$, $\dot{x}_1$-$\dot{x}_2$) calculated for $m_1=0.03$ kg, $m_2=0.38$ kg, $k_1=0.16$ kN/m, $c=0.03$ Ns/m, $k_2=221$ kN/m, $\mu =0.5$, $G=7$ mm, $\Omega =5$ Hz, and $P_{d}$=1.81 N. The location of the impact boundary is shown by the vertical blue line

**Fig. 12:**
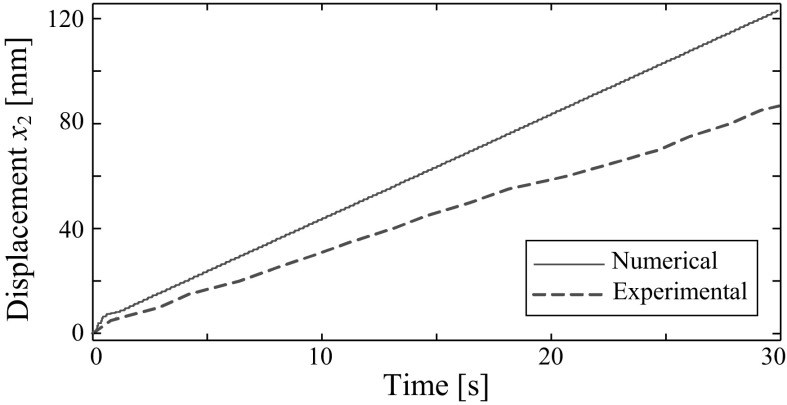
Comparison of numerical and experimental displacements of the capsule for $m_1=0.03$ kg, $m_2=0.38$ kg, $k_1=0.16$ kN/m, $c=0.03$ Ns/m, $k_2=221$ kN/m, $\mu =0.5$, $G=7$ mm, $\Omega =5$ Hz, $P_{d}$=1.81 N, $P_{v}=5$ V, and $P_{o}=0$ V

The numerical result calculated using the parameters in Table 2, $k_2=221$ kN/m, $\Omega =5$ Hz, and $P_{d}=1.81$ N is shown in Fig. 11. Here, $P_{d}$ was approximated by using the specification of the solenoid provided by the manufacturer, i.e. the solenoid can generate a maximum of 1.81 N force for a stroke of 7 mm if the amplitude of excitation is 5 V. It can be seen from the figure, the capsule system has a period-one motion with three impacts per period of excitation, and the capsule progresses forward gradually once the impact between the rod and the plate occurs. The experiment was carried out by using the same parameters above with $P_{v}=5$ V and $P_{o}=0$ V, and the displacement of the capsule is shown with the numerical result in Fig. 12. Comparing both numerical and experimental displacements, a quantitative discrepancy can be observed, which might be due to the inaccurate estimation of the friction coefficient. However, both capsule progressions are forward consecutively for 30 seconds.

**Fig. 13:**
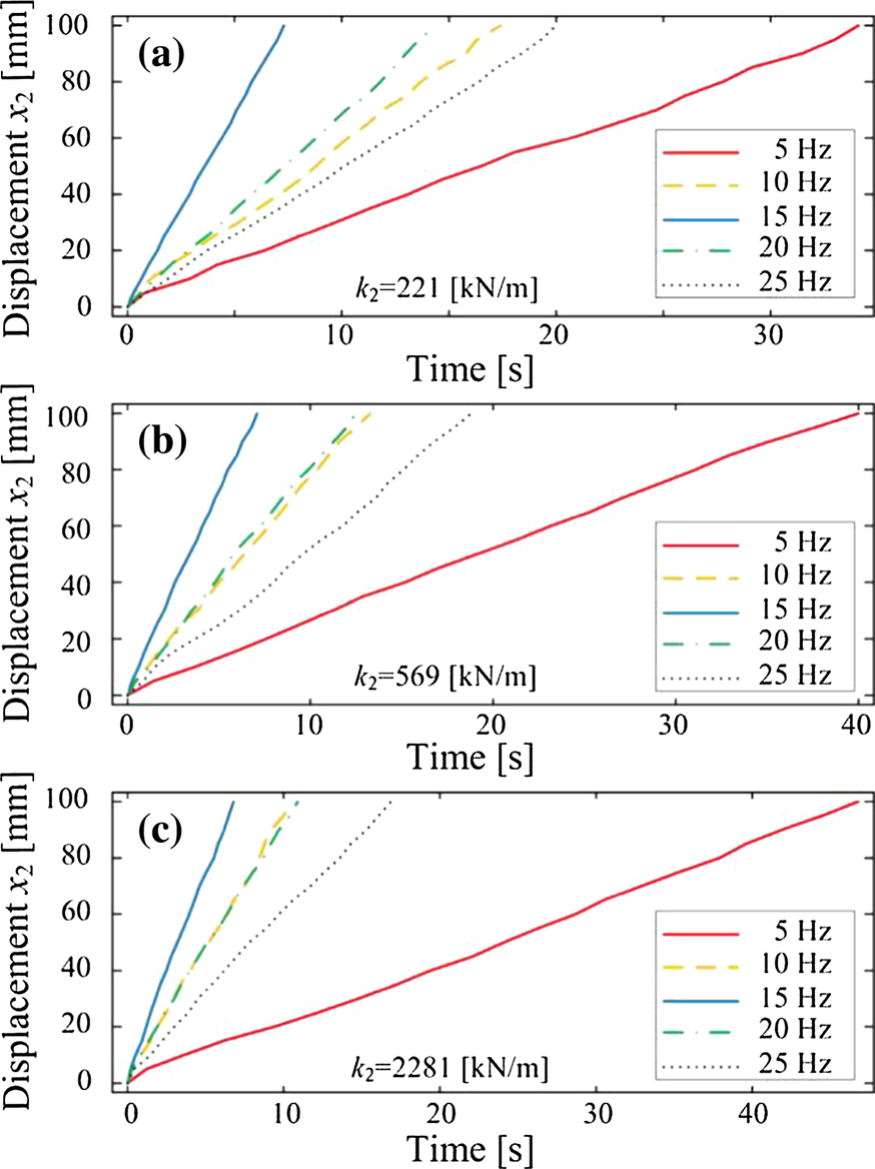
Experimental results: time histories of displacements of the capsule obtained for $m_1=0.03$ kg, $m_2=0.38$ kg, $k_1=0.16$ kN/m, $c=0.03$ Ns/m, $\mu =0.5$, $G=7$ mm, $P_{v}=5$ V, $P_{o}=0$ V, **a** $k_2=221$ kN/m, **b** $k_2=569$ kN/m, and **c** $k_2=2281$ kN/m

Figure 13 shows the results of experimental investigation under variations of the secondary stiffness $k_2$ and the frequency of sinusoidal excitation $\Omega$. All the experiments were run for a total distance of 100 mm and the excitation frequency was tested between 5 and 25 Hz. Three different values of the secondary stiffness were adopted by changing the overhang length of the impact plate. As can be seen from the figure, the capsule excited at 15 Hz is much faster than the one excited at 5 Hz, and the speeds of the capsule excited at 10 and 20 Hz become similar when the stiffness of the plate increases.

**Fig. 14:**
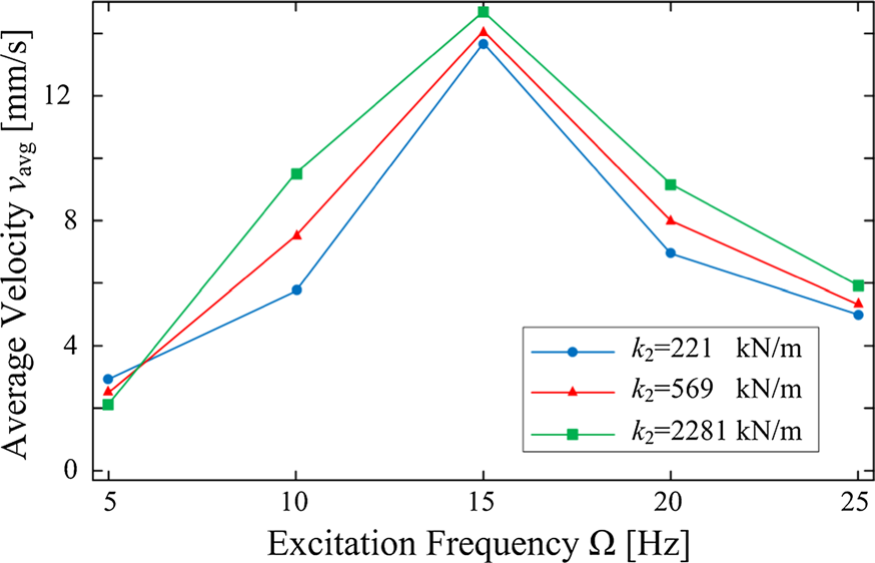
Average velocities of the capsule recorded for $m_1=0.03$ kg, $m_2=0.38$ kg, $k_1=0.16$ kN/m, $c=0.03$ Ns/m, $\mu =0.5$, $G=7$ mm, $P_{v}=5$ V, and $P_{o}=0$ V

In order to compare the capsule speed in terms of the stiffness of the plate, we calculated the average velocity of the capsule using $v_{avg}=X_2/\,T_c$, where $X_2$ is the total displacement of the capsule, $T_c$ is the total time consumption, and the corresponding results are plotted in Fig. 14. As can be observed from the figure, except the results for 5 Hz, the capsule moves faster when the stiffness of the plate is larger. At 5 Hz, experimental results show that the lower stiffness, the faster average progression. For the best capsule progression, it was achieved by $\Omega =15$ Hz and $k_2=2281$ kN/m, which gave the capsule an average speed at 14.7 mm/s.

**Fig. 15:**
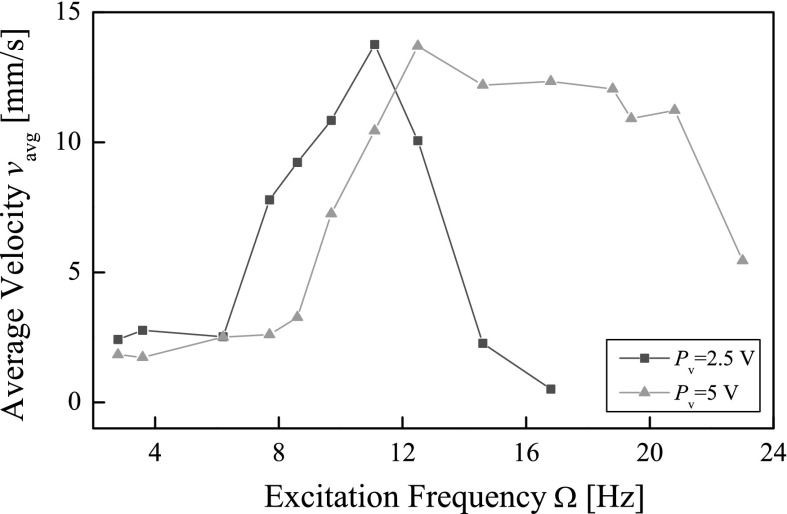
Average velocities of the capsule recorded for $m_1=0.03$ kg, $m_2=0.38$ kg, $k_1=0.16$ kN/m, $c=0.03$ Ns/m, $k_2=2281$ kN/m, $\mu =0.5$, $G=7$ mm, and $P_{o}=0$ V

**Fig. 16:**
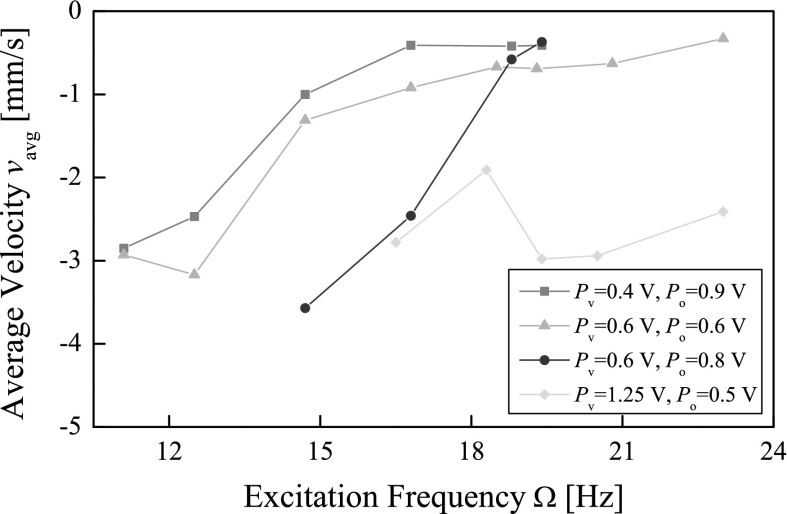
Average velocities of the capsule recorded for $m_1=0.03$ kg, $m_2=0.38$ kg, $k_1=0.16$ kN/m, $c=0.03$ Ns/m, $k_2=2281$ kN/m, $\mu =0.5$, and $G=7$ mm

Extensive experiments were carried out under variations of excitation offset $P_{o}$. In fact, the change of the offset may affect the gap between the rod and the plate. However, for $P_{o}=0$, the gap remains at the original value, $G=7$ mm. Each test was run for 300 mm, and the recorded average velocities of the capsule are plotted in Figs. 15 and 16. It can be seen from Fig. 15 that, when $P_{o}=0$, the best forward progression was achieved at $\Omega =11.1$ Hz and $\Omega =12.5$ Hz for $P_{v}=2.5$ V and $P_{v}=5$ V, respectively. Comparing with the experimental results shown in Fig.  14, it is found that for the amplitude of excitation $P_{v}=5$ V, the frequencies for the best capsule progression are slightly different, which is due to the fact that the later experiment took a longer measurement distance for calculating the average progression of the capsule. It should also be noted that, for $P_{v}=2.5$ V, the average progression of the capsule decreases drastically when the excitation frequency increases after the best frequency at $\Omega =11.1$ Hz.

Various amplitude and offset of the sinusoidal excitation were tested, and the results are presented in Fig. 16, where only backward progressions of the capsule are shown. It can be observed that, the best backward progression of the capsule was achieved at $\Omega =14.7$ Hz, $P_{v}=0.6$ V, and $P_{o}=0.8$ V, and the backward average speed decreases as the excitation frequency increases.

## Conclusions

The prototype development of the vibro-impact capsule system for pipeline inspection was studied in this paper. The capsule is driven by a periodically excited inner mass which intermittently impacts with a weightless plate connected to the capsule through a linear spring. The system can progress forward or backward once the net force on the capsule overcomes its external resistance. Numerical simulations were carried out using non-smooth system modelling approach in order to reveal the fundamental mechanism of the system.

Base on the physical model of the capsule system, the prototype was designed using SolidWorks and manufactured through 3D printing. The prototype employs a push-type solenoid as the actuator, and the rod of the solenoid intermittently impacts a clamped steel plate, which leads to the progression of the entire system. For sinusoidal excitation of the rod, digital-to-analog converter and Arduino UNO board were used to convert discrete numerical numbers into continuous voltage. Remote control of the system was also developed using the radio frequency wave. Two transceiver modules were built to implement the communication between the control panel and the capsule. By using the wireless module, the frequency, the amplitude, and the offset of the sinusoidal excitation can be controlled in real-time.

The capsule was tested in a dry uPVC clear pipe and the experimental results were compared with numerical simulations. Although there is a quantitative discrepancy between the simulation and the experiment due to the inaccurate estimation of the friction coefficient, both capsule progressions show the same forward trend. Extensive experiments were carried out by varying the stiffness of the plate, the frequency, the amplitude, and the offset of the sinusoidal excitation. Both forward and backward progressions of the capsule were observed, and their optimum control parameters for the best average progression were obtained.

Future works include experimental testing of the prototype in a fluid environment, geometric optimization of the capsule for the minimum drag and lift forces, integration of inspection sensors (e.g. ultrasonic nondestructive testing sensor), and design of position tracking system for the capsule. In addition, another perspective of the work is the mathematical modelling of the capsule system with multiple modules, e.g. [13, 30, 31]. It is possible to further develop the capsule model studied in this paper to realize a more complex motion, such as a planar or a three-dimensional motion. Research findings along this direction will be reported in due course.
